# Circuits for State-Dependent Modulation of Locomotion

**DOI:** 10.3389/fnhum.2021.745689

**Published:** 2021-11-10

**Authors:** Alejandro J. Pernía-Andrade, Nikolaus Wenger, Maria S. Esposito, Philip Tovote

**Affiliations:** ^1^Institute of Clinical Neurobiology, University Hospital Würzburg, Würzburg, Germany; ^2^Department of Neurology, Charité – Universitätsmedizin Berlin, Corporate Member of Freie Universität Berlin and Humboldt Universität zu Berlin, Berlin, Germany; ^3^Medical Physics Department, Centro Atomico Bariloche, Comision Nacional de Energia Atomica, Consejo Nacional de Investigaciones Cientificas y Tecnicas, San Carlos de Bariloche, Argentina; ^4^Center for Mental Health, University of Würzburg, Würzburg, Germany

**Keywords:** circuits and circuit components, motor control, neural networks, gait, emotional states, locomotion

## Abstract

Brain-wide neural circuits enable bi- and quadrupeds to express adaptive locomotor behaviors in a context- and state-dependent manner, e.g., in response to threats or rewards. These behaviors include dynamic transitions between initiation, maintenance and termination of locomotion. Advances within the last decade have revealed an intricate coordination of these individual locomotion phases by complex interaction of multiple brain circuits. This review provides an overview of the neural basis of state-dependent modulation of locomotion initiation, maintenance and termination, with a focus on insights from circuit-centered studies in rodents. The reviewed evidence indicates that a brain-wide network involving excitatory circuit elements connecting cortex, midbrain and medullary areas appears to be the common substrate for the initiation of locomotion across different higher-order states. Specific network elements within motor cortex and the mesencephalic locomotor region drive the initial postural adjustment and the initiation of locomotion. Microcircuits of the basal ganglia, by implementing action-selection computations, trigger goal-directed locomotion. The initiation of locomotion is regulated by neuromodulatory circuits residing in the basal forebrain, the hypothalamus, and medullary regions such as locus coeruleus. The maintenance of locomotion requires the interaction of an even larger neuronal network involving motor, sensory and associative cortical elements, as well as defined circuits within the superior colliculus, the cerebellum, the periaqueductal gray, the mesencephalic locomotor region and the medullary reticular formation. Finally, locomotor arrest as an important component of defensive emotional states, such as acute anxiety, is mediated via a network of survival circuits involving hypothalamus, amygdala, periaqueductal gray and medullary premotor centers. By moving beyond the organizational principle of functional brain regions, this review promotes a circuit-centered perspective of locomotor regulation by higher-order states, and emphasizes the importance of individual network elements such as cell types and projection pathways. The realization that dysfunction within smaller, identifiable circuit elements can affect the larger network function supports more mechanistic and targeted therapeutic intervention in the treatment of motor network disorders.

## Introduction

As animals evolved to adapt to highly dynamic environments, they developed nervous systems that supported a large arsenal of scaled behavioral responses to varying stimuli and contexts. Adequate action selection thus became dependent on complex internal states, capable of dynamically controlling specific motor patterns. Consequently, higher organisms may initiate movements relying on cognitive or emotional reference (Takakusaki, 2017). However, regardless of whether the driver of the movement is volitional or emotional, goal-oriented locomotion requires body postural control which includes balance adjustment and muscle tone regulation (Grillner, 1975). Pioneering studies implementing selective spinal cord and brain-region lesions in cats identified the spinal cord as the locus for the control of the step cycle (i.e., stance and swing, left and right alternation), usually referred to as central pattern generator (Grillner, 2003). Seminal studies identified three brain regions underlying the supraspinal control of locomotion, the DLR (originally referred to as the subthalamic locomotor region), the MLR, and the CLR (Shik and Orlovsky, 1976; Grillner, 2003). A reticulospinal excitatory network within the brainstem locomotor center was hypothesized as the ultimate supraspinal station producing locomotor patterns, in close interaction with sensory feedback (Grillner, 2003). Based on the organizational principle of functionally distinct brain areas, our knowledge on how the brain controls movements greatly improved throughout the following decades. While the region-specific function concept reflects important determinants of brain function, including motor control, the advent of combined genetic and optical methodologies in basic neuroscience has recently added the perspective of a brain-wide neuronal network (Ferreira-Pinto et al., 2018). This network consists of microcircuits interconnected by long-range projection pathways forming functional modules. In this system that is dependent on both, the hardwired microcircuits and their long-range interconnections, as well as the dynamic information flow within them, somatosensory information and emotions interact at different levels in high-order brain areas to orchestrate action selection from initiation to termination of locomotion. Therefore, gait dysfunction needs to be looked at from a network perspective. In this review, we aim to integrate both views by describing the large-scale interactions among brain areas for cognition, defense and movement as interactions of defined circuit elements that are required for the state-dependent modulation of gait.

### Categorization of Locomotion

How do we address and operationalize complex state-dependent modulation of specific movement functions? It has been proposed that locomotion can be divided into three behavior-relevant categories, exploratory locomotion, primary appetitive locomotion and primary defensive locomotion. Such categories are regulated by the hypothalamus and the preoptic area of the BF of rats (Sinnamon, 1993), suggesting that emotions play a central role in the regulation of locomotor region functions and may guide moment-to-moment changes in exploratory or defensive states in an animal. Circuits-centric behavioral research has shown that exploratory and appetitive/consummatory locomotion rely mainly on the circuitry formed among the BF, the hypothalamus and BG (Sinnamon, 1993), whereas, defensive locomotion engages the orchestrated action of defensive circuits involving the amygdala, the hypothalamus and the periaqueductal gray (PAG) (LeDoux, 2012). On the other hand, early experiments in decerebrated cats indicate that the three locomotor regions have well defined roles for the initiation of movements, such that DLR-lesioned animals are unable to perform goal-driven locomotion but they are able to perform coordinated walking and running upon MLR stimulation (Shik and Orlovsky, 1976). Conversely, animals with cerebellar ablation can not walk by themselves but once the body position is assisted (e.g., head fixed and body suspended in a hammock) they can perform uncoordinated locomotion upon stimulation of DLR and MLR. This evidence suggests that volitional locomotion relies on DLR, the coordination of locomotion requires CRL, whereas executive locomotion relies on MLR. However, whether the emergence of a specific behavioral state (e.g., exploration, hunting or defensive behavior) requires the activation of one or several locomotor regions and whether the locomotor regions cooperate or compete to favor a specific behavioral outcome are still open questions.

While the categorization of locomotion based on the behavioral context directly points to the regulatory role of higher-order states, locomotion can also be differentiated more descriptively into initiation, maintenance and termination phases, temporal dynamics that are tightly linked to gait function (Sinnamon, 1993; Ferreira-Pinto et al., 2018). This approach supports the view that these motor phases and resulting locomotor patterns are not *per se* defined by a certain state, but represent basic motor programs accessible and modulated by higher-order states. Consequently, we will review experimental evidence dissecting the neural basis of state-dependent modulation of initiation, maintenance and termination of locomotion as well as discuss their relevance in gait function.

## Initiation of Locomotion

### Cellular Identity of Locomotor Initiation Drivers

**FIGURE 1:**
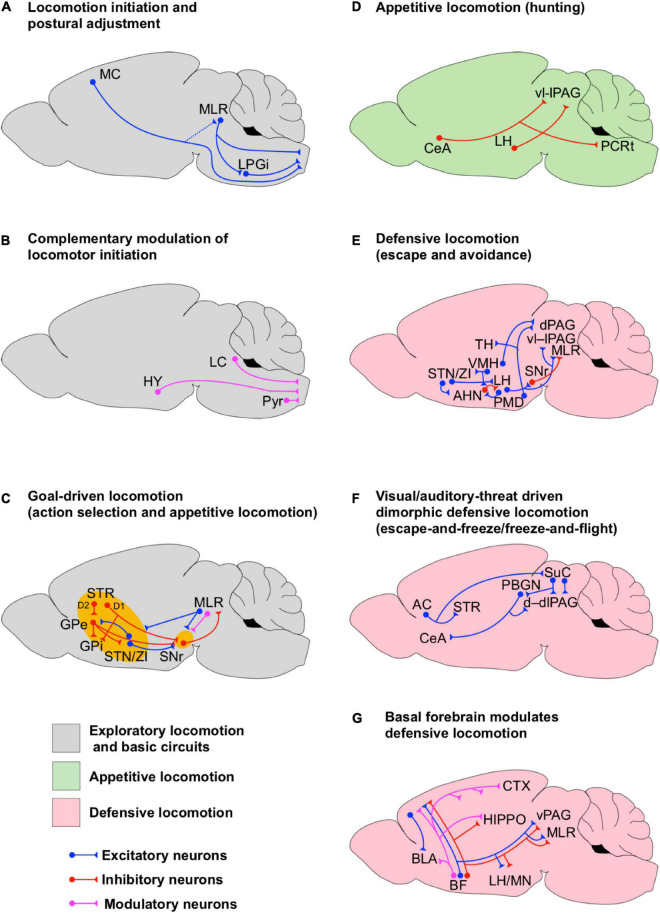
Circuits for state-dependent initiation of locomotion. **(A)** Scheme of the basic neuronal circuits underlying the initiation of locomotion. The dotted line between the motor cortex (MC) and the MLR denotes minor monosynaptic contacts between both areas in rodents. **(B)** Scheme of the neuronal circuits underlying the complementary modulation of the initiation of locomotion. **(C)** Scheme of the neuronal circuits underlying the initiation of goal-driven locomotion. These circuits account for action selection and appetitive locomotion. Note the interconnections between BG and MLR. The BG circuits are highlighted in yellow to indicate that they are involved in the initiation of a particular state even if they are not fully shown for simplicity. **(D)** Scheme of the neuronal circuits underlying the initiation of appetitive locomotion. **(E)** Scheme of the neuronal circuits underlying the initiation of defensive locomotion. **(F)** Scheme of the neuronal circuits underlying the initiation of dimorphic defensive locomotion (escape-and-freeze and freeze-and flight). **(G)** Scheme of the basal forebrain neuronal circuits underlying the modulation of defensive locomotion.

Although classic electrical microstimulation studies have identified three regions in the brain capable of eliciting locomotion, MLR has been investigated the most using new genetic tools for the dissection of cell-type specificity. Light-induced stimulation of individual MLR neuronal subtypes demonstrated that glutamatergic activation is sufficient to induce locomotion from rest (Roseberry et al., 2016; Capelli et al., 2017; Caggiano et al., 2018; Josset et al., 2018), whereas stimulation of cholinergic neurons positively modulates the speed of ongoing locomotion (Roseberry et al., 2016). Conversely, among other studies, former research showed that electrical stimulation of the PPN, where cholinergic neurons reside, evokes atonia and induces rapid-eye movements in decerebrate cats (Takakusaki et al., 2004, 2005) suggesting that PPN hosts a strikingly complex neural network able to modulate motor responses supporting different brain states. Later on, Capelli et al. (2017) found that optogenetic activation of glutamatergic neurons in the LPGi of the medullary reticular formation was: (1) sufficient to initiate forward-directed full-body locomotion of mice in an open-field arena, and was (2) necessary for high-speed locomotion evoked by MLR stimulation (Roseberry et al., 2016; Capelli et al., 2017; Caggiano et al., 2018; Josset et al., 2018; Carvalho et al., 2020). This initiation of locomotion was restricted to LPGi glutamatergic neurons as similar stimulation of subnuclei adjacent to the reticular formation failed to initiate locomotion. These results provide direct evidence of an excitatory brainstem neuronal network underlying the initiation of locomotion (Figure 1A). However, it has recently been demonstrated that glutamatergic MLR neuronal subpopulations fulfill functional roles that extend far beyond the control of locomotion (Garcia-Rill et al., 1986; Sherman et al., 2015; Roseberry et al., 2016; Chang et al., 2020; Ferreira-Pinto et al., 2021). Strikingly, glutamatergic MLR neurons with descending projections to the spinal cord (Figure 1A) are tuned to full body behaviors such as rearing and locomotion, whereas glutamatergic MLR neurons with ascending axonal terminals impinging to BG output regions are tuned to forelimb behaviors such as handling and grooming. Both neuronal subpopulations are intermingled within the PPN and the adjacent mesencephalic reticular region (mRt) and can only be disentangled by their projection specificity. Moreover, gain- and loss-of-function experiments demonstrated the functional specificity of descending spinally projecting glutamatergic MLR neurons for body extension during rearing and the initiation of locomotion. In contrast, optogenetic manipulation of ascending glutamatergic MLR neurons resulted in a more generalized modulation of body movements (Ferreira-Pinto et al., 2021). The observation that opposing functions coexist within such small brain areas may explain controversial results on PPN function by previous studies showing a role of glutamatergic PPN neurons in low-speed exploratory locomotion, locomotion arrest or both (Roseberry et al., 2016; Capelli et al., 2017; Caggiano et al., 2018; Josset et al., 2018; Carvalho et al., 2020).

The initiation of fine and skillful locomotion is one of the functions of the MC (Grillner, 2003; Kawai et al., 2015; Dhawale et al., 2021), however, MLR may also complement MC through the modulation of brainstem premotor circuits (Esposito et al., 2014). Other studies have also shown that the activation of noradrenergic (alpha2), dopamine (D1/D2), and serotonin (5-HT2 and 7) receptors in the spinal cord is sufficient to initiate and maintain locomotion in intact and strikingly, even spinalized laboratory animals (Smythe and Pappas, 1989; Giroux et al., 2001; Jordan et al., 2008; Cregg et al., 2020). The selective activation of these receptors has been shown to modulate the spinal somatosensory-motor network, the step kinematics and the left-right limb coordination. Anatomical evidence indicates that the spinal noradrenergic afferents originate at the LC (Li et al., 2016), the dopaminergic afferents originate at the hypothalamus (Qu et al., 2006), whereas serotonin afferents originate at the parapyramidal region of the medulla oblongata (Jordan et al., 2008). Overall, excitatory circuit elements within the MLR play a central role in the initiation of locomotion, which is complemented by modulatory biogenic amines (Figures 1A,B).

### Basal Ganglia Circuits as a Functional Module for the Initiation of Goal-Directed and Exploratory Locomotion

The striatum and STN receive topographically organized synaptic projections from several cortical motor and limbic association areas including primary MC, dorsal and ventral premotor cortices, supplementary motor area, and the rostral, dorsal and ventral portions of cingulate motor areas (DeLong and Wichmann, 2007). Therefore, BG is a central hub for the integration and execution of cortical information. Strikingly, while MC is essential for the acquisition of timed instrumental motor skills, the expression of these motor program kinematics, once learned, no longer relies on the MC but on the directly connected subcortical circuits of the dorsolateral striatum (Kawai et al., 2015; Dhawale et al., 2021). Extensive research on BG circuitry has shown that the initiation of goal-oriented locomotion, instrumental learning and its reinforcement happen through the activation of two BG synaptic pathways between the striatum and the main output areas, GPi and the substantia nigra pars reticulata (SNr) (Yin et al., 2005; DeLong and Wichmann, 2007; Kravitz et al., 2012; Freeze et al., 2013). One BG pathway, referred to as direct pathway, is made by monosynaptic inhibitory afferents from the SPNs in the dorsal striatum to GPi and SNr. The second BG pathway, referred to as indirect pathway, is made by a disynaptic disinhibitory projection from the dorsal striatum to the STN, GPi, and SNr via GPe (Figure 1C). Thus, activation of inhibitory SPNs from the indirect pathway will suppress the inhibitory control of GPe on STN, GPi, and SNr. Since STN is interconnected to the hypothalamus, it is possible that GPe functions as a gate for the emotional trigger of locomotion in order to initiate exploration or consummatory actions. However, deeper research on GPe as an emotional motor gate is needed.

Striatal projection neurons in the striatum involved in the BG indirect pathway express inhibitory dopamine receptors type 2 (D2) while SPNs involved in the BG direct pathway express excitatory dopamine receptors type 1 (D1) (Neve et al., 2004). Thus, dopamine release in the striatum by afferents from the substantia nigra pars compacta (SNc) exerts a differential modulation on both pathways, characterized by the suppression of the indirect path while facilitating the inhibitory influence of the direct path on GPi/SNr neurons (Figure 1C). Since STN sends excitatory projections to GPi and SNr, the temporal interplay and balance between the direct and indirect pathways as well as the functional organization of GPi/SNr neuronal engrams may determine the output of BG. SNr and GPi provide differential axonal projections to several components of the thalamocortical and brainstem motor systems turning BG into a central broadcaster for motor control (Kooy and Carter, 1981; McElvain et al., 2021). Interestingly, the GPi/SNr neurons have been shown to provide tonic inhibition to motor thalamocortical neurons and neural circuits in PPN (Grillner et al., 2005, 2008; DeLong and Wichmann, 2007). However, selective optogenetic stimulation of either the direct or indirect pathways *in vivo* generates both excitation and inhibition of subpopulations of neurons in SNr, although, the effectiveness of direct pathway stimulation in producing movement initiation is correlated with inhibited subpopulations of SNr neurons (Freeze et al., 2013). Conversely, effective indirect pathway-mediated motor suppression has been shown to be most strongly influenced by excited SNr neurons (Freeze et al., 2013). Former research has also shown a segregated effect of the activation or either pathway on gait functions during ambulation (Kravitz et al., 2010). Kravitz et al. (2010) found that the bilateral optogenetic excitation of the indirect pathway decreases locomotor initiation, increases immobility and promotes bradykinesia, whereas the activation of the direct pathway increases locomotion and reduces immobility. Moreover, these effects were mediated by MLR activity (Roseberry et al., 2016; Capelli et al., 2017; Caggiano et al., 2018; Josset et al., 2018; Figure 1C). Altogether, these data show that BG promotes movement by an overall disinhibition of downstream targets.

Further studies implementing selective optogenetic inhibition or excitation of either direct or indirect BG pathways *in vivo* (D1– or D2–type driven opsin expression respectively) has shown that there is a complementary interaction between both pathways for the initiation and maintenance of goal-driven consummatory actions (Tai et al., 2012; Tecuapetla et al., 2016; Yttri and Dudman, 2016). Optogenetic stimulation of the BG direct pathway in dorsomedial striatum (associative area) of mice biases the initiation of learned consummatory actions toward the contralateral site, whereas optogenetic stimulation of the BG indirect path does it for the ipsilateral site (Tai et al., 2012). However, the bias is effective only if the stimulation happens before the animal initiates motor actions. Furthermore, the stimulation of each pathway also increases the reaction time (latency) to a “Go” signal suggesting that both pathways cooperate in the decision-making process. Interestingly, outside the decision-making task, the stimulation of the direct path is able to facilitate contralateral motion while the stimulation of the indirect path is ineffective suggesting that the direct path, but not the indirect one, is also involved in body postural adjustment and locomotion direction control. Once the consummatory action has been initiated, the velocity of the forepaw to activate a reward system seems to reinforce the velocity of future motor consummatory actions in a positive or negative way depending on whether the direct path or the indirect path has been simultaneously stimulated in the dorsomedial striatum respectively (Yttri and Dudman, 2016). Surprisingly, the forepaw-velocity triggered optical stimulation does not affect the rate of motion initiation and reward consumption, suggesting a dissociation between the forepaw motor dynamics and a cognitive action-selection.

**FIGURE 2:**
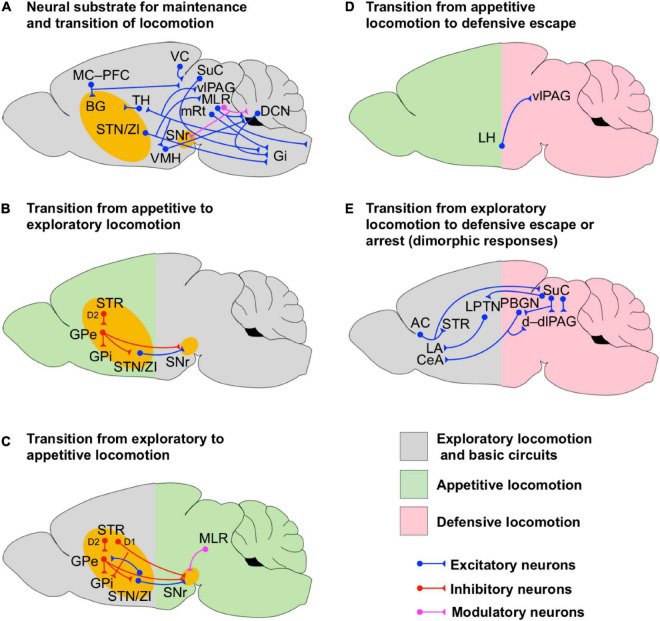
Circuits for transition and maintenance of locomotion. **(A)** Scheme of the neuronal circuits underlying the maintenance and transition of locomotion. The intricate network among the cerebellum, BG, hypothalamus, PAG, MLR and their projections to Gi are shown. **(B,C)** Scheme of the BG neuronal circuits supporting the bidirectional transition from appetite to exploratory locomotion **(B)** and exploratory to appetite locomotion **(C)**. Notice that the transition from exploratory to appetitive locomotion is supported by feedback modulatory projections from MLR. The BG circuits are highlighted in yellow to indicate that they are involved in the initiation of a particular state even if they are not fully shown for simplicity. **(D)** Scheme of the neuronal circuits underlying the transition from appetitive to defensive locomotion. **(E)** Scheme of the neuronal circuits underlying the transition from exploratory locomotion to dimorphic defensive escape or arrest. Notice that the dimorphic defensive behaviors are driven by visual or auditory sensory inputs routed via the superior colliculus (SuC) and auditory cortex (AC) respectively.

Other study indicates that the effect of optogenetic stimulation of the BG pathways on decision-making tasks is not restricted to the dorsomedial striatum as the stimulation of these pathways in the dorsolateral striatum (sensorimotor area) also produce comparable results on the latency and the reward consumption (Tecuapetla et al., 2016). However, the effect in the dorsolateral striatum is not reinforced as seen in the dorsomedial striatum (Kravitz et al., 2012). Tecuapetla et al. (2016) also showed that the decision-making process is not restricted to SPNs but also engages parvalbumin-positive (PV+) GABAergic interneurons in the striatum. Notably, stimulation of the indirect path, but not the direct path, interrupts the maintenance of consummatory behavior and favors ambulation (exploratory locomotion; Figure 2B), suggesting that the indirect path in the dorsolateral striatum is involved in the transition of behavioral states whereas the direct path seems to be engaged in action initiation.

All together, these results suggest that the BG pathways complement each other to form a functional module which supports goal-driven motor performance. In this BG module both pathways seem involved in the decision-making process (e.g., action selection), however, at the action execution level, the direct path seems to support the initiation of motor actions, postural adjustment and locomotion direction while the indirect path does it for the maintenance and the termination of goal-driven behaviors (state transition initiation, Figure 1C).

### Interactions of Globus Pallidus and Hypothalamus With the Subthalamic Nucleus Mediate State-Dependent Behavioral Effects

Malfunction or damage of the striatal network may generate an imbalance in the locomotor control and maladaptive behavior. For instance, compulsive grooming in rodents appears to be a consequence of a reduced tonic inhibition on SPNs in the centromedial/dorsomedial striatum (Burguière et al., 2013, 2015). In normal conditions, such a tonic inhibition counterbalances the excitatory input from the neocortex to SPNs. Moreover, downstream from the striatum (Figure 1C), selective optogenetic activation of GPe GABAergic interneurons or GPe-projecting STN afferents produces hyperkinesia and abnormal involuntary movements in mice, such as abnormal forelimb posture, neck’s torsion spasm, and compulsive grooming, chewing and licking (Tian et al., 2018). Noteworthy, the activity of STN neurons is also regulated by the hypothalamus, hence, compulsive behaviors may be triggered by altered emotional states as well.

Early experiments in anesthetized rats indicated that the hypothalamus modulates the firing rate of STN/ZI neurons (Narita et al., 2002). Narita et al. (2002) showed that activation of KARs in the VMH, which is part of the neural circuitry underlying the initiation of the defensive behavior (Sinnamon, 1993; LeDoux, 2012), increases the firing rate of STN/ZI neurons (Figure 1E). Interestingly, in another set of experiments these authors showed that activation of KARs in VMH increases the locomotion speed in rats walking on a running wheel, which could be due to the activation of defensive neural circuits. Importantly, the running speed was reduced by micro-injections of KARS antagonists or GABA (natural agonist of inhibitory GABA receptors) in STN/ZI, suggesting that they have a pivotal role in the initiation of defensive locomotion.

Anatomical evidence indicates that ZI projects to the AHN and to the LH (Mitrofanis, 2005), both of which are interconnected (Figure 1E) and work together in the initiation of defensive locomotion (Canteras, 2002; Wang et al., 2015; Li et al., 2018). Interestingly, a subset of excitatory LH projection neurons that co-releases glutamate and the neuropeptide orexin on their postsynaptic targets has been shown to facilitate the initiation, but not the maintenance, of locomotion in freely moving mice in a way that it is proportionally modulated by the hunger state of the animal (Karnani et al., 2020). Furthermore, studies in decerebrate cats have shown that microinjection of orexin in CNF, PPN and SNr has facilitatory effects on locomotion (Takakusaki et al., 2005). Orexin injected in CNF directly increases MLR-electrically evoked locomotion by lowering the current threshold needed to evoke locomotion, whereas orexin injected in PPN and SNr reduces atonia associated with PPN-electrically induced rapid-eye movements. However, this last effect is reversed by subsequent injection of bicuculline (a GABA_A_ receptor antagonist) in PPN, indicating that elevation of inhibitory synaptic transmission in PPN is needed for the induction of atonia. Moreover, activation of PPN inhibitory synapses counterbalances the pro-locomotion effect of orexin on PPN and SNr. Former research in anesthetized and acutely decerebrated cats indicates that SNr provides an important inhibitory synaptic control on PPN neurons needed for the induction of atonia (Takakusaki et al., 2004). The evidence presented in this section indicates how specific circuit elements within GP, SNr, STN/ZI, and hypothalamus may interact to initiate locomotion and mediate state-dependent behavioral effects (Figures 1D,E). Malfunction of the neural circuits residing in these areas may favor the emergence of maladaptive behaviors.

### The Basal Forebrain, a Modulatory Cholinergic Center Wiring High-Order Brain Functions to the Initiation of Locomotion

Early studies implementing selective microinjection of glutamate or picrotoxin (GABA_A_-receptor blocker) in the BF in anesthetized rats indicate that the activation of postsynaptic excitatory glutamate receptors and reduction of GABAergic inhibition in different BF nuclei elicit stepping (Sinnamon, 1993). How does it happen? This is still an open question, however, one possible explanation is that the initiation of locomotion in these physiological preparations may be due to the activation of downstream areas associated with motor or defensive circuits. BF contains intermingled populations of GABAergic, glutamatergic and cholinergic neurons which regulate a number of different brain functions such as arousal, memory, learning and defensive responses. This happens through the modulation of neuronal excitability and synaptic function in thalamus, cortex, hippocampus, and amygdala (Steriade et al., 1993; Vogt and Regehr, 2001; Rogers and Kesner, 2003; Sarter et al., 2003; Hasselmo, 2006; Henny and Jones, 2008; Hasselmo and Stern, 2014; Lin et al., 2015; Unal et al., 2015; Jiang et al., 2016; Gielow and Zaborszky, 2017; Howe et al., 2017). Subpopulations of non-cholinergic BF neurons encode salience, reward and punishment information to regulate learning and decision making (Lin et al., 2015). However, the modulation of learning not only relies on BF glutamatergic and GABAergic projections to the neocortex but also relies on the BF cholinergic projections to a broader range of cortical areas and the hippocampus, which also receives BF GABAergic projections (Rogers and Kesner, 2003; Sarter et al., 2003; Hasselmo, 2006; Henny and Jones, 2008; Hasselmo and Stern, 2014; Agostinelli et al., 2019). Cholinergic subpopulations of BF neurons regulate defensive neuronal circuits and associated behavioral responses via projections to the amygdala (Mark et al., 1996; Picciotto et al., 2012; Unal et al., 2015; Jiang et al., 2016). Namely, optogenetic activation of modulatory cholinergic BF projections to the BLA increases the encoding signal-to-noise ratio in BLA principal neurons and enhances glutamatergic synaptic transmission within the BLA, which favors the induction of long-term-potentiation of cortical-amygdalar synapses (Unal et al., 2015; Jiang et al., 2016). Whether subpopulations of BF neurons encoding for salience, reward and punishment are directly engaged in the modulation of defensive behavioral responses is still unknown. However, Jiang et al. (2016) have shown that the acquisition of fear memory depends on the activation of BF cholinergic projections to the BLA. BF also sends glutamatergic and GABAergic projections to a number of subcortical areas, including defensive circuits in the hypothalamus and PAG as well as circuits in MLR (Swanson et al., 1984; Agostinelli et al., 2019). The evidence presented above indicates that BF may affect defensive learning and locomotion via the modulation of an intricate network between cognitive, defensive and mesencephalic locomotor circuits (Figure 1G).

Extensive evidence has also shown that BF cholinergic neurons degenerate in different cognitive and motor neurodegenerative diseases in humans, such as Alzheimer’s disease, Lewy Bodies dementia, atypical Parkinsonian’s diseases (PD), alcoholic dementia and Parkinson’s disease (Pepeu et al., 2015), indicating the central role of the BF modulatory system in the regulation of cognitive and motor functions in the human brain. The evidence presented in this section indicates that BF has a central role in the regulation of cognitive and motor actions via the modulation of distinct brain areas involved in the processing of high-order cognitive functions and emotions. However, while deterioration of the BF neurons has been associated with slow gait and falls in PD patients (Bohnen et al., 2019) the precise neuronal path underlying the initiation of locomotion remains elusive.

### The Hypothalamus, Amygdala and Periaqueductal Gray Contain Circuits for the Initiation of Appetitive and Defensive Locomotion

**FIGURE 3:**
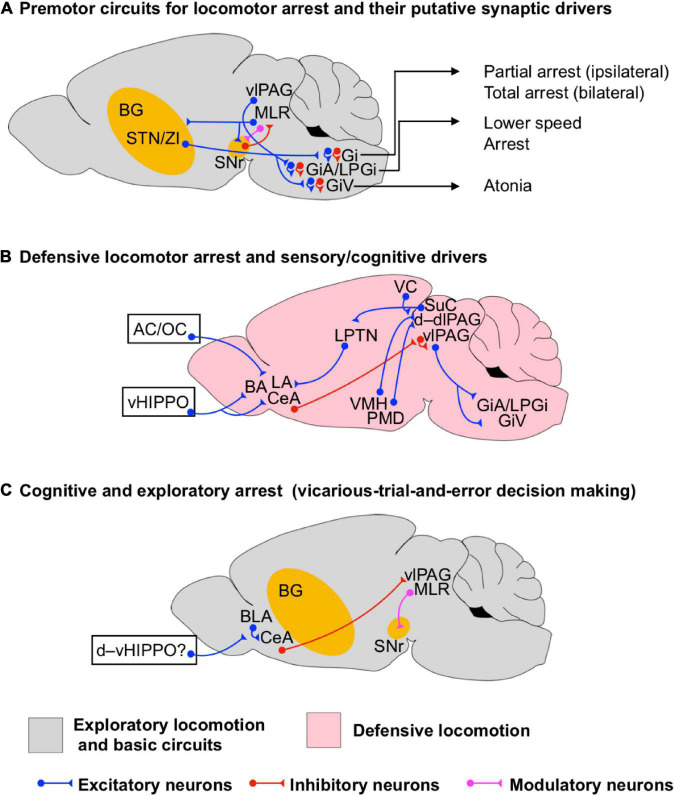
Circuits for locomotor arrest during exploratory and defensive states. **(A)** Scheme of the premotor neuronal circuits underlying locomotor arrest and their putative synaptic drivers. Different types of locomotion arrest executed by premotor neuronal circuits in the medullary reticular formation are indicated (arrows). **(B)** Scheme of the neuronal circuits underlying the initiation of defensive locomotor arrest. Sensory and cognitive synaptic drivers are shown such as from auditory cortex (AC), olfactory cortex (OC), and the ventral hippocampus (vHIPPO). **(C)** Scheme of the neuronal circuits underlying cognitive arrest during spatial exploration, such as that seen during exploratory decision making. Potential synaptic drivers from the dorsoventral hippocampus (d–vHIPPO) involved in non-defensive spatial memory processing are shown. The question mark indicates that the actual synaptic path from d–vHIPPO to BLA/CeA underlying decision-making-driven arrest has not been identified. The BG circuits are highlighted in yellow to indicate that they are involved in the initiation of a particular state even if they are not fully shown for simplicity.

A major hub for modulation of appetitive/consummatory and defensive locomotion is the hypothalamus (Sinnamon, 1993; Canteras, 2002; LeDoux, 2012). The hypothalamus in turn provides major monosynaptic excitatory and inhibitory neuronal projections from different subregions to PAG (Figures 1E, 2D,E, 3B; Vianna and Brandão, 2003; Motta et al., 2009; Keay and Bandler, 2015; Li et al., 2018). Early anatomical studies using chemical retrograde and anterograde tracing revealed that such projections are differentially distributed along the dorsoventral anatomical subdivisions of PAG sharing input areas with excitatory afferents from the auditory and visual sensory cortices, the anterior cingulate cortex and the retrosplenial cortex at the dorsal, dorsolateral and lateral PAG columns (d, dl, and lPAG, respectively), with afferents from the rostral prelimbic cortex at the ventral and ventrolateral PAG (v, vlPAG, respectively), with afferents from the fore- and hindlimb motor cortices at the v, vl, and lPAG and with diffuse afferents from the dorsal raphe nucleus (Vianna and Brandão, 2003). Other evidence shows that dPAG also receives monosynaptic glutamatergic projections from the SuC (Figure 1F; Evans et al., 2018). In addition, the vlPAG receives inhibitory monosynaptic projections from amygdala via CeA (Keay and Bandler, 2015; Tovote et al., 2015; Figure 1D). The integration of hypothalamic, amygdalar, cortical and collicular synaptic inputs by PAG neurons may provide high-order correlated emotional, cognitive and sensory information to be delivered to MLR and medullary premotor neurons. Notably, recent evidence indicates that PAG is also involved in the modulation of arousal (Porter-Stransky et al., 2019) complementing the actions of LC (Aston-Jones and Cohen, 2005). Namely, norepinephrine released from LC afferents increases glutamatergic synaptic transmission onto vPAG-DA neurons, as a consequence wakefulness is increased. Arousal modulates the activity of primary visual cortex neurons by enhancing visual encoding and reducing noise correlation (Vinck et al., 2015), which may improve the quality of visual information delivered to PAG and associative cortices, for instance. Interestingly, arousal seems to complement locomotion in the modulation of the activity of primary visual cortex neurons (Vinck et al., 2015), such a modulation may help to tune the visual information processed during exploratory and goal-driven behaviors.

Selective optogenetic activation of inhibitory (GABAergic) and excitatory (glutamatergic) projections from LH to the ventrolateral and lateral areas of PAG (vl–lPAG) in mice has been shown to drive predation and threat evasion respectively (Figures 1D,E, 2D; Li et al., 2018). Li et al. (2018), using projection specific optogenetic manipulation and fiber photometry *in vivo*, demonstrated that LH GABAergic neurons become transiently active when a mouse starts attacking a prey but remain silent during prey consumption. Li et al. (2018) also showed that optogenetic inhibition of LH GABAergic cell bodies or their afferents on vl–lPAG suppresses predatory behavior. Conversely, optogenetic stimulation of LH GABAergic afferents in vl–lPAG is sufficient to increase the attack probability. These results suggest that these LH GABAergic–vl–lPAG circuits are engaged in the initiation of appetitive locomotion such as hunting (Figure 1D). Furthermore, a recent study suggests that hunting behavior driven by LH GABAergic neurons occurs through the inhibition of vl–lPAG neurons involved in the facilitation of defensive behaviors like flight and cornering (protective arrest) for instance (Rossier et al., 2021). Rossier et al. (2021) realized that during prey recognition, which requires several approaches, the mice are in a defensive state (showing defensive signs), then, once the actual prey is identified the mice turn into a predatory mode. These authors found that optogenetic stimulation of LH GABAergic afferents in the vl–lPAG (Figure 1D) during early prey recognition time (dominated by defensive behavior) reduced attack latency and increased attack persistence while reducing cornering and escape. Furthermore, stimulation of LH GABAergic fibers reduced the activity of vl–lPAG neurons associated with defensive and exploratory investigation behavior (sniffing environment). Interestingly, stimulation of LH GABAergic somas in settled predators not only increased the attack performance but also increased compulsive biting without changing food consumption. These results suggest that LH GABAergic neurons are sufficient to drive hunting behavior partially by suppressing the activity of pro-defensive and exploratory neurons in vl–lPAG, however, LH GABAergic neurons also favor aggressive and compulsive behavior.

Other evidence suggests that predatory behavior is also complemented by the inhibitory projections from CeA on to vl–lPAG (Figure 1D; Han et al., 2017). Han et al. (2017) suggested that CeA commands a modular system to drive predatory hunting. This modular system is made by subpopulations of CeA GABAergic neurons projecting to vl–lPAG and to the parvicellular reticular formation (PCRt). The activation of the vl–lPAG pathway increased the stalking time on the prey and hunting velocity while reducing latency to hunt and capture duration. On the other hand, the CeA-PCRt pathway increased mastication and tuned the postural muscles of the neck to facilitate feeding. The coexistence of the LH and CeA systems suggest that an optimal hunting performance requires the integration of emotional and sensory information (via amygdala and hypothalamus) at the vl–lPAG and postural control and feeding via PCRt.

Li et al. (2018), also showed that vl–lPAG projecting LH glutamatergic neurons are directly related to the initiation of evasive behaviors (Figure 2D). Activation of these neurons caused mice to immediately cease food retrieval and to start running and jumping in the opposite direction. On the other hand, inhibition of this pathway reduces, but not abolishes, the escape responses to actual physical threats, suggesting that this LH-vl–lPAG path is not the only one supporting the transition from appetitive to defensive escape. Another study has shown that a single hypothalamic nucleus is able to initiate opposing behaviors depending on its postsynaptic targets. Optogenetic activation of glutamatergic neurons in the dorsomedial and central areas of VMH (dm/cVMH) promotes avoidance to a safe environment with increased locomotion (Figure 1E) but at the same time facilitates immobility (Wang et al., 2015). Wang et al. (2015) showed that these opposing behavioral effects are due to the fact that, on one hand, dm/cVMH neurons form functional circuits with GABAergic neurons in AHN to promote avoidance and increase locomotion (Figure 1E), but on the other hand, it forms circuits with dlPAG to facilitate immobility (Figure 3B). Interestingly, dm/cVMH has subpopulations of neurons projecting exclusively to either area and another subpopulation projecting to both areas, however, the specific natural contextual trigger activating each pathway is unknown. The results of Wang et al. (2015) agree with the observation of an increased running speed in rats after the microinjection of kainate in VMH (Narita et al., 2002). However, Narita et al. (2002) also showed that such a VMH motor effect is sensitive to the pharmacological manipulation of STN/ZI. We discussed before that ZI projects to AHN and LH (Figure 1E; Mitrofanis, 2005). In LH, a subset of excitatory orexinergic neurons that co-releases glutamate and orexin on CNF, PPN and SNr facilitates the initiation of locomotion in behaving mice (Figure 1E; Karnani et al., 2020) and in decerebrate cats (Takakusaki et al., 2005). Hence, it is possible that the initiation of locomotion induced by dm/cVMH also engages the neuronal paths ZI–AHN/LH to modulate the activity of CNF, PPN, and/orSNr neurons (Mitrofanis, 2005; Takakusaki et al., 2005; Karnani et al., 2020). However, further research must be done to identify the specific neuronal subpopulations involved in the initiation of defensive locomotion. Recent evidence has also shown that ZI is directly connected to executive premotor excitatory neurons in Gi involved with the control of locomotion direction (Cregg et al., 2020) and to glutamatergic neurons in PPN and CNF (Roseberry et al., 2016; Capelli et al., 2017; Caggiano et al., 2018; Josset et al., 2018). Therefore, further research should explore the neural circuits within and among hypothalamus, MLR and other brainstem motor regions to see how they contribute to the initiation of locomotion in a broader range of behavioral states.

Recent evidence indicates that dPAG also receives excitatory projections from CCK-expressing glutamatergic neurons residing in the PMD (Wang et al., 2021). These CCK-expressing PMD neurons favor defensive escape (Figure 1E). Interestingly, a subpopulation of these neurons projecting to the thalamus are also activated during context-specific escape that requires spatial navigation (Figure 1E). The dPAG also receives visual information from SuC via monosynaptic glutamatergic projections (Figure 1F). This pathway provides salience threatening visual cues that defines a synaptic threshold for dPAG activation and the initiation of escape (Evans et al., 2018). Subpopulations of PV+ excitatory projection neurons in SuC have been shown to initiate dimorphic defensive behaviors such as escape and freezing when an animal is exposed to visual environmental threats such as looming visual stimuli (Shang et al., 2015, 2018). Such neurons do not project directly to the PAG but they do to the parabigeminal nucleus (PBGN) and the LPTN (Figures 1F, 2E, 3B). LPTN projects to LA (Doron and LeDoux, 2000) while PBGN projects to CeA (Usunoff et al., 2006; Shang et al., 2015) and dlPAG (Meller and Dennis, 1986; Klop et al., 2006). Activity within the SuC-LTPN-LA pathway has been shown to trigger freezing (Shang et al., 2015, 2018; Wei et al., 2015) whereas, PBGN has been shown to initiate a behavioral pattern of escape-and-freeze responses (Shang et al., 2018). Strikingly, both pathways compete against each other to initiate a specific behavioral outcome (escape-and-freeze or freeze alone), as optical inactivation of PBGN favors freezing while optical inactivation of LPTN favors escape-and-freeze. However, the precise neural mechanism underlying the natural balance between PBGN and LTPB to control the expression of dimorphic defensive behaviors via amygdala and dlPAG is still an open question. Recently, looming auditory cues have also been shown to initiate complex behavioral defensive sequences, such as freeze-and-flight (Li et al., 2021). The underlying network comprises divergent projections from the AC onto striatal D2-SPNs and SuC neurons (Figure 1F). Finally, although it is not explicitly discussed in this review, we acknowledge that putative excitatory circuits between the ventrolateral area of VMH and d–dlPAG underlie aggressive defense (e.g., fight) (Hashikawa et al., 2016), which may also contribute to the initiation of defensive locomotion (Figure 1E). However, extensive research on this pathway is still needed.

In light of the evidence presented in this section, we hypothesize that the initiation of goal-driven, appetitive, and defensive locomotion relies on well-defined and distinct functional neuronal modules (Figures 1, 2D,E), which may work together in a context-dependent manner. While MC supports fine motor control, the MLR is involved in postural adjustments as well as high and low speed locomotion (Figure 1A). Moreover, modulatory projections from LC, the hypothalamus and the parapyramidal region of the medulla oblongata to the spinal cord support the initiation and maintenance of locomotion (Figure 1B). Furthermore, BG supports general goal-driven locomotion, action selection and associative learning (e.g., Pavlovian and instrumental learning; Figure 1C) and it can also be influenced by appetitive and defensive information via the hypothalamic projection to STN (Figure 1E). The initiation of appetitive locomotion is supported by vlPAG which integrates synaptic information from the hypothalamus and the amygdala (Figure 1D). Interestingly, PAG seems to have a special role in the integration and sorting of synaptic information conveying either appetitive or defensive signals (Figures 1E,F), having its more dorsal domains (i.e., d–dlPAG) engaged mainly in active defensive locomotion, whereas the more ventral domains (i.e., l–vlPAG) are involved in appetitive as well as active and passive defensive responses. Information of active defense, such as escape and flight, is conveyed by the synaptic inputs from several specific hypothalamic nuclei (Figure 1E) and the SuC (Figure 1F). Interestingly, it has been shown that glutamatergic CNF neurons are necessary for escape responses triggered by an air-puff (Roseberry et al., 2016; Capelli et al., 2017; Caggiano et al., 2018; Josset et al., 2018). Whether and how defense circuits interact with glutamatergic CNF neurons to trigger active responses remains to be addressed. Finally, the BF is involved in a broad range of brain functions, but it may support defensive locomotion via the modulation of several defensive circuits and its direct projection to MLR (Figure 1G).

## Maintenance and Coordination of Locomotion

### Basic Network Control of Locomotion Rhythm and Direction

Coordination of rhythmic locomotion and gait rely to a large extent on the activity of spinal circuits in vertebrates (Sinnamon, 1993; Grillner, 2003). However, in order to contribute to goal-driven locomotion, these spinal circuits must be activated by supraspinal locomotor centers (Grillner et al., 2008; Cregg et al., 2020). Excitatory neurons in the MLR are directly responsible for setting locomotion speed and gait patterns (Figures 1A, 2A; Roseberry et al., 2016; Capelli et al., 2017; Caggiano et al., 2018; Josset et al., 2018). For instance, high-speed synchronous locomotion is mediated by CNF glutamatergic neurons while PPN glutamatergic and cholinergic neurons contribute to low-speed locomotion and arrest (Roseberry et al., 2016; Capelli et al., 2017; Caggiano et al., 2018; Josset et al., 2018). The three DCN (Figure 2A) in the CLR, such as the FN, the IN and the DN, interact to govern posture, locomotion, fine finger movements, gaze, the acquisition of Pavlovian oculomotor conditioning and refined complex computational prediction-error processes underlying the encoding and reinforcement of fear memories (Bostan et al., 2013; Vitale et al., 2016; Hintzen et al., 2018; Wang et al., 2018; Cregg et al., 2020; Frontera et al., 2020). Such a diverse set of functions of the cerebellum relies on the synaptic interactions with vlPAG, PPN, the pontine and medullary premotor neuronal nuclei, and the thalamic ventrolateral nucleus (Figure 2A). Interestingly, the activity of Purkinje cells at the cerebellar cortex and neurons in FN and IN are modulated by the stepping rhythm (Orlovsky, 1972a,b) suggesting that the cerebellum is directly involved in sensing rhythmic locomotion. FN is directly involved with the control of postural muscles and locomotion (Mori et al., 1998) and together with the DN projects to excitatory premotor neurons in Gi (Figure 2A; Cregg et al., 2020). Neurons of FN have also been shown to project to the vlPAG (Figure 2A) targeting excitatory (Chx10-positive), inhibitory (GAD2-positive), and modulatory (TH-positive) neurons (Vaaga et al., 2020). However, optical stimulation of FN afferents has controversial effects on Chx10-positive neurons. On one hand, FN optical stimulation produces an artificial postsynaptic depolarization of Chx10-positive neurons which brings these neurons to fire trains of action potentials. On the other hand, such an optical stimulation not only induces a postsynaptic reduction of the amplitude of evoked excitatory currents on Chx10-positive neurons but also induces a postsynaptic increase of the amplitude of evoked inhibitory synaptic currents on these neurons via the activation of dopamine receptor type 2 (D2) *in vitro*. Since activation of vlPAG glutamatergic projection neurons to the magnocellular nucleus of the medulla promotes locomotion arrest (Tovote et al., 2016; Figure 3B), FN may favor locomotion by enhancing synaptic inhibition on these neurons, however, whether the selective stimulation of FN afferents *in vivo* favors or not locomotion arrest is still an open question. The evidence presented above indicates that the cerebellum is a neural hub involved in the processing of information in defensive and sensorimotor circuits (Figure 2A), hence, it may have a pivotal role in the general control of locomotion.

Cregg et al. (2020) found that optogenetic activation of Gi glutamatergic Chx10-positive neurons (Figure 2A) causes inhibition of ipsilateral rhythmic locomotion activity and excitation (contraction) of ipsilateral axial muscles in mice. Such a dual action bends the mouse body trunk on the ipsilateral site, reduces the ipsilateral flexor-locomotor activity and force generation, and promotes ipsilateral turning. Conversely, unilateral inhibition of Chx10-positive neurons facilitates contralateral turning. The unilateral motor effect of Chx10-positive neurons in Gi is due to the activation of spinal inhibitory circuits as it is sensitive to the pharmacological blocking of spinal inhibitory synaptic transmission. Further anatomical studies with transsynaptic tracers showed that Chx10 Gi neurons receive synaptic inputs from the contralateral SuC, ipsilateral ZI, ipsilateral mRt and from the deep cerebellar nuclei DN (bilateral) and IN (ipsilateral), suggesting that Chx10 Gi neurons integrate somatosensory and motor information from the DLR and the CLR to drive spinal motor circuits and evoke turning (Figure 2A). Surprisingly, Chx10 Gi neurons do not receive synaptic projections from MLR (Figures 2A, 3A) suggesting a greater complexity of the neural network underlying locomotion initiation, maintenance and direction. While initiation mainly requires LPGi (collecting MLR information), maintenance also requires Gi (collecting DLR and CLR information). Interestingly, Cregg et al. (2020) also showed that mice do not have the ability to compensate for the dysfunction of the Gi-Chx10 turning system which suggests that deterioration of the Gi-Chx10 network or its supraspinal drivers may have direct consequences on gait function. The evidence presented before suggests that the premotor circuits in Gi are critical for the postural adjustment needed for the maintenance of locomotion (Figure 2A).

### The Cerebellum and the Basal Ganglia as a Hub for the Multilevel Integration of Sensory-Motor, Defensive and Cognitive Information

Maintenance of locomotion and gait requires the coordination of circuits underlying sensorimotor integration and computation of higher-order brain functions. It is thought that the cerebellar and BG circuits manage the integration of sensorimotor and cognitive information supporting anticipatory postural adjustment and gait function. This integration happens through reciprocal connections with the frontal-parietal and motor cortices, brainstem, and spinal sensory-motor circuits (Koutsikou et al., 2015; Takakusaki, 2017; Frontera et al., 2020). Both BG and cerebellum are interconnected through the disynaptic loops DN–thalamus–dorsal striatum and STN–PPN–DN–cerebellar cortex (Figure 2A; Bostan et al., 2013; Vitale et al., 2016). However, the determination of the specific roles of these circuits in the maintenance and coordination of locomotion is still a challenge. One possible explanation of this difficulty is that the activation of PPN neurons produces different effects on their postsynaptic targets and induces opposing behavioral responses. For instance, electrical microstimulation of PPN enhances the cerebellar output by directly increasing the activity of DCN neurons in rats (Vitale et al., 2016). Such a neuronal effect is mediated by cholinergic and glutamatergic PPN projections, however, the actual behavioral consequence of the PPN-evoked excitation of DCN neurons remains elusive. Other evidence indicates that electrical microstimulation of PPN induces atonia in decerebrate cats which depends on the elevation of inhibitory synaptic transmission in PPN (Takakusaki et al., 2004, 2005). Further evidence indicates that the ascending PPN-cholinergic neuronal projections onto SNr provide direct inhibition to striatal direct pathway axonal terminals, which results in a reduction of the velocity of locomotion (Moehle et al., 2017). Conversely, other results showed that optogenetic activation of cholinergic PPN neurons positively modulates ongoing locomotion while the activation of glutamatergic neurons in PPN favors low-speed exploratory locomotion and locomotion arrest (Roseberry et al., 2016; Capelli et al., 2017; Caggiano et al., 2018; Josset et al., 2018; Carvalho et al., 2020). Since the same putative cell type can generate opposite behavioral responses, we hypothesize that the output of PPN depends on the dynamic interaction of competing glutamatergic, cholinergic and GABAergic neuronal engrams supporting the maintenance and coordination of locomotion in a context dependent manner.

As mentioned before, the cerebellum (Figure 2A) participate in a broad range of motor and cognitive brain functions (Bostan et al., 2013; Vitale et al., 2016; Hintzen et al., 2018; Wang et al., 2018; Cregg et al., 2020; Frontera et al., 2020). Furthermore, evidence shows that the ascending sensory and proprioceptive information traveling to the cerebellum via the spino-olivary pathways is under the modulatory control of PAG, which seems to provide a selective filter for balancing the nociceptive and proprioceptive information (Koutsikou et al., 2015). Likewise, Koutsikou et al. (2015) indicated that PAG also modulates the cerebellar output. The described modulation of sensory input and output of the cerebellum by PAG activity suggests that defensive circuits may play a very important role in the modulation of cerebellar functions. Anatomical and electrophysiological studies in rats have shown that the cerebellum is also interconnected with the hypothalamus (Figure 2A; Supple, 1993; Onat and Çavdar, 2003). The anterior cerebellar vermis receives projections from the ventrolateral hypothalamus (VMH/LH area). Supple and collaborators reported that stimulation of VMH/LH generates a transient increase-decrease sequence in the firing rate of putative Purkinje cells. On the other hand, back projections from the cerebellum via FN neurons have also been reported to arrive at the posterior and the dorsomedial hypothalamic nuclei (Newman and Reza, 1979; Onat and Çavdar, 2003). However, the functional role of these projections on cerebellar or autonomic functions is unknown. As aforementioned, the hypothalamus plays a direct role in the initiation of defensive locomotion via interactions with the BG circuits (Figure 1E; Narita et al., 2002; Mitrofanis, 2005; Takakusaki et al., 2005; Karnani et al., 2020), therefore, the cerebellum might be also involved in the initiation of defensive locomotion. Together, the evidence presented in this section suggests that the cerebellum-BG network provides a neural system that supports different types of locomotion (Figure 2A). Moreover, this system has multiple points of interaction with the defensive circuits via PAG and hypothalamus, allowing its emotional or defensive modulation (Figures 1E, 2A).

### Cholinergic Counterbalance of the Dopaminergic Control of Basal Ganglia Output

The output of BG is modulated by the activation of cholinergic neurons in the striatum and PPN facilitating the reduction of locomotion upon the exposure to a contextual reward (Figures 1C, 2A; Picciotto et al., 2012; Moehle et al., 2017). The striatal cholinergic neurons pause their firing (Goldberg and Reynolds, 2011) while the PPN cholinergic neurons increase their firing upon the exposition to salient reward-related cues (Picciotto et al., 2012). Tonically active striatal cholinergic interneurons increase neural excitability of SPNs and suppress feed-forward excitatory and feed-back inhibitory synapses onto SPNs (Oldenburg and Ding, 2011). This happens via activation of the muscarinic acetylcholine receptors type M1 and M2. The tonic cholinergic control of the striatal network may also support adaptive adjustment in the processing of sensory information as it undergoes different forms of synaptic plasticity (Oldenburg and Ding, 2011; Davis et al., 2018; Abudukeyoumu et al., 2019). On the other hand, PPN cholinergic neurons provide direct inhibition to the synaptic terminals of D1-expressing SPNs arriving at the SNr (Figures 1C, 2A) via activation of the muscarinic receptor type M4 (Moehle et al., 2017). While the striatal cholinergic modulation may provide a mechanism for the direct computation of sensory information by SPNs, the PPN cholinergic modulation is more in charge of the direct regulation of the BG output. This evidence also suggests that the PPN-striatal cholinergic system may counterbalance the pro-locomotion effect of dopamine in a context–dependent manner, which may facilitate the transition from high-speed locomotion to slow locomotion and arrest.

### Locomotor State Transitions Support Adaptive Behavior

Transitions between distinct locomotion states are required for switching between adaptive behaviors when an organism copes with environmental and situational challenges. The correct communication within a global network supports optimal evaluation and selection of action options such as exploratory, appetitive and defensive locomotion. Evidence presented in the former sections indicates that the activation of the indirect pathway of BG facilitates the transition from consummatory to exploratory behavior (Figure 2B; Tecuapetla et al., 2016). Upon presentation of salient reward-related cues, cholinergic PPN neurons increase their activity (Picciotto et al., 2012). These neurons project back to SNr where they inhibit the direct BG pathway input to slow down and arrest locomotion (Moehle et al., 2017). This system may facilitate the transition between exploration to consummatory behavior (Figure 2C). Upon a potential threat, LH-glutamatergic neurons projecting to vlPAG terminate food retrieval and promote defensive escape (Figure 2D; Li et al., 2018). Escape can be also complemented by the activation of the dm/cVMH-AHN pathway (Figure 1E; Wang et al., 2015). Furthermore, VMH may also increase the activity of STN/ZI neurons (Narita et al., 2002) augmenting the weight of information in the BG indirect pathway which may lead to reduction of speed or may bias goal-driven behavior (Tai et al., 2012; Tecuapetla et al., 2016). However, how the VMH-STN/ZI pathway modulates goal-driven locomotion is still unknown. Higher-order state regulation involves neurons in the basal amygdala, which predict the transition between exploratory, non-exploratory, and defensive behavioral states (Gründemann et al., 2019). Finally, dimorphic defensive behaviors such as escape-and-freeze orchestrated by the SuC-PBGN/LPTN networks (Figure 2E; Shang et al., 2018) and freeze-and-flight managed by the cortico-striatal and cortico-collicular networks (Li et al., 2021) are examples of the capacity of the sensory system to drive transitions between behavioral states. Overall, transitions from one to another locomotion state, although ultimately resulting in stereotyped behavioral and gait patterns, require activity within specific circuit elements depending on the context, stimulus and internal state of the organism.

Based on the evidence presented in the previous subsections, we hypothesize that the maintenance and coordination of locomotion rely on the precise temporal interaction among neuronal modules in the cerebellum, BG, hypothalamus, amygdala, PAG and MLR (Figure 2). Remarkably, BG seems to be self-sufficient to drive the bidirectional transition between appetitive and exploratory locomotion, although the regulation of locomotion speed is supported by MLR (Figures 2B,C). Sensory information arriving to the brain via sensory cortices and the cerebellum, may be then routed to BG, TH, and PAG (Figures 2A,E). Motor information processed by MC and BG is then transmitted to PAG and MLR (Figures 2A–C,E). In PAG, the sensory/motor information may be integrated with appetitive and defensive information arriving from hypothalamus and amygdala (Figures 1E, 2D). In MLR/mRt, the information may be sent back to BG via ascending feedback projections (Figures 1C, 2A,C) for further processing and to medullary LPGi and Gi neurons via descending projections (Figures 1A, 2A) to initiate locomotor transitions. In turn, Gi may integrate and compute motor commands from mRt, cerebellum, BG and PAG to send postural-adjustment commands to the spinal motor circuits, while LPGi may provide to the spinal cord the trigger command for the initiation of locomotion (Figure 1A).

## Termination of Locomotion

While termination of locomotion, strictly speaking, only describes the moment in which gait is stopped, it is tightly linked to immobility, which in many instances follows termination of locomotion. In the threatening contexts, this immobility is commonly termed freezing, originally defined as the absence of all movements despite respiration. To capture termination of locomotion in various contexts and across different states, we will use the more descriptive term arrest, which conceptually includes termination of locomotion and subsequent immobility (Figure 3).

### Basic Network Underlying the Termination of Locomotion

Different to the initiation and maintenance of rhythmic locomotion, the basic neural circuitry underlying the termination of locomotion is more diversified. Bouvier et al. (2015) found that bilateral optogenetic activation of Chx10 Gi glutamatergic neurons results in locomotor arrest by inhibiting the rhythmic motor activity in the spinal cord (Figure 3A). Later on, Cregg et al. (2020) found that the unilateral activation of the same neurons halts the ipsilateral rhythmogenesis, facilitating directional turning during exploratory locomotion. In addition, Capelli et al. (2017) found that optogenetic activation of glycinergic neurons in the medullary reticular formation elicits different forms of locomotion arrest (Figure 3A). Whereas stimulation of LPGi and GiA glycinergic neurons is sufficient to reduce speed and halt locomotion without affecting posture, GiV neurons provoked body collapse resembling behavioral atonia, and Gi neurons also produced body collapse and spasms. These results suggest that distinct functional forms of locomotor arrest are mediated by the activation of different subpopulations of neurons located in the hindbrain. Other evidence shows that the direct inhibitory control of MLR glutamatergic neurons encoding locomotor state and speed by BG circuits and/or activation of local GABAergic MLR neurons is necessary and sufficient to terminate locomotion (Roseberry et al., 2016). Furthermore, optogenetic activation of a glutamatergic MLR neuronal subpopulation identified by its ascending projection to BG output regions evoked halting of locomotion as well as other behaviors (Garcia-Rill et al., 1986; Sherman et al., 2015; Roseberry et al., 2016; Chang et al., 2020; Ferreira-Pinto et al., 2021). All together, this evidence suggests that the termination of locomotion relies on the dynamics of the MLR–BG network, and hindbrain circuits including GiA, GiV, LPGi and Gi (Figure 3A). However, whether specific circuits are recruited to drive context- or state-dependent locomotor arrest remains to be determined.

### Higher Order Network Elements for the Termination of Locomotion

Next to a role in locomotion initiation, the hypothalamus also plays a central role for its termination. As mentioned before, the postsynaptic activation of the VMH network increases the firing rate of STN/ZI neurons (Narita et al., 2002), as a consequence, the BG indirect pathway may suppress exploratory locomotion (Kravitz et al., 2010; Freeze et al., 2013). Moreover, Wang et al. (2015) found that selective optogenetic activation of steroidogenic-factor 1 (SF1)-expressing neurons in dm/cVMH projecting to dlPAG produces arrest (Figure 3B). Stimulation of dm/cVMH also produces autonomic responses resembling the development of behavioral stress such as pupil dilation, increase in breathing rhythm and in heart rate. However, Wang et al. (2015) also reported that the changes of autonomic responses are dissociated from active locomotion as they happen during freezing behavior, suggesting that defensive arrest is functionally associated with an elevation of stress.

Motta et al. (2009) have shown that neurons in the PMD (Figure 3B) become differentially activated by distinct contextual threats (i.e., conspecific and predatory threats) and the downstream PAG responds differentially to these threats as well. Analysis of the activation of the early gene c-fos at PAG revealed that dominant conspecific threats activate neurons in PAG in a way that follows the axonal projections from the ventrolateral region of PMD (vlPMD, where c-fos is upregulated by the intruder), whereas a predatory threat generates a c-fos pattern that follows the projections from the dorsomedial region of PMD (dmPMD, where c-fos is upregulated by the predator). Exposure to a predator increases c-fos activity mainly in d–dlPAG, whereas exposure to a dominant conspecific not only increases c-fos at d–dlPAG but also at lPAG. However, the increase of c-fos at lPAG may also be due to the correlated activation of sensory cortical and collicular inputs for instance (Shang et al., 2018; Li et al., 2021). Furthermore, whether the correlated conspecific-predator responses between PMD and d–lPAG rely on the activation of PMD-CCK-expressing glutamatergic neurons (Wang et al., 2021) is still an open question. Analysis of defensive reactions in PMD-lesioned mice showed that defensive arresting behaviors (i.e., freezing and playing dead) were reduced while active defensive behaviors (i.e., standing upright position, boxing and fleeing) were unaffected in reference to control animals. These results suggest that the dmPMD/vlPMD–d,dl,lPAG circuits are specially involved in the facilitation of defensive behavioral arrest.

The defensive neural circuits involved in the processing of auditory, visual, and olfactory sensory stimuli can elicit defensive arrest (Figure 3B), presented as a single behavioral sign or in complex behavioral sequences such as freeze-and-flight (Euston et al., 2012; Lisman et al., 2017; Rozeske et al., 2018). Using optogenetic manipulations of specific cell types, single-unit recordings and rabies-mediated neuroanatomical tracings, Tovote et al. (2016) dissected a pathway from CeA to the vlPAG that mediates freezing by disinhibition of the vlPAG glutamatergic output to descending premotor neurons in the magnocellular nucleus of the medulla (i.e., LPGi, GiA and GiV) (Figure 3B). Inhibition of glutamatergic PAG neurons greatly attenuated freezing behavior both to learned and innate threats. However, whether the excitation of these premotor neurons also engages the activation of GiA-, and/or LPGi-glycinergic neurons to promote defensive arrest remains elusive. Later on, Xu et al. (2016), by using trans-synaptic viral tracing and optogenetic manipulations, found that the vHIPPO (a central component of circuits processing emotions and contextual memory) and the amygdala interact via multiple parallel pathways (Figure 3B). Projections from subsets of vHIPPO to the basal amygdala mediates the retrieval of context-dependent freezing (after fear extinction), whereas a parallel projection from a distinct subset of vHIPPO neurons onto CeA neurons projecting to the vlPAG is necessary for context-dependent renewal of cued fear memories. These results suggest that the activation of parallel circuits between vHIPPO and the amygdala underlies the behavioral expression of high-order cognitive functions such as the retrieval and renewal of contextual memory leading to defensive locomotor arrest. Other evidence suggests that the neural circuits between vHIPPO and the amygdala also support the behavioral expression of non-defensive spatial memory, which is a natural function of the dorsal hippocampus in rodents (Lisman et al., 2017; Figure 3C). Using behavioral analyses, circuit mapping, single-cell calcium imaging and closed-loop optogenetic approaches, Botta et al. (2020) identified cell ensembles in BLA whose activation was correlated with momentary pauses (∼1 s) in exploratory locomotion. Usually the arrests were followed by changes in the angular speed of the head resembling the movements made by rodents while performing vicarious-trial-and-error decision making (Figure 3C; Redish, 2016). This suggests that during such an arrest the animal may be going through a cognitive processing to evaluate the spatial options. Interestingly, optogenetic activation of CeA-projecting BLA neurons decreases locomotion and promotes arrest while inhibition of glutamatergic BLA neurons facilitates movements. Furthermore, the BLA neuronal ensembles are spatially modulated as they become reactivated when the animals revisit familiar locations (i.e., habituation-home area and the boundaries of an open field maze). However, whether the described non-defensive behavioral arrest engages CeA-vlPAG projections or not still needs to be demonstrated.

Hippocampus and the medial PFC (mPFC) work together in the processing of associative memory and learning. While HIPPO is involved in encoding and early consolidation, mPFC is involved in late consolidation and the development of schematic representations of cognitive tasks and emotional contexts (Dejean et al., 2016; Karalis et al., 2016). More specifically, the dorsomedial PFC (dmPFC) is directly involved in contextual fear discrimination by the dynamic neural representation of threatening and non-threatening contexts (Rozeske et al., 2018; Bagur et al., 2021). Rozeske et al. (2018), demonstrated that subpopulations of l–vlPAG-projecting neurons in dmPFC (Figure 2A) have the property to dynamically represent both threatening and non-threatening multisensory contexts. This occurs by increasing their firing rate in non-threatening contexts. Thus, the activity of this subpopulation of dmPFC neurons is inversely correlated with freezing. Furthermore, optogenetic activation of dmPFC afferents at l–vlPAG reduces freezing while inhibition favors it. However, whether the effect of dmPFC relies on the dopaminergic modulation of the Chx10-positive neurons at l–vlPAG (Vaaga et al., 2020) remains to be determined. Interestingly, other studies indicate that the frequency-modulated synchronization between dmPFC and the amygdala is essential for the initiation of contextual freezing (Li et al., 2021). Moreover, synaptic dynamics and neuronal firing of fear-related dmPFC neurons and the maintenance of contextual freezing appears to be modulated by breathing-related neuronal activity of the olfactory bulb (Bagur et al., 2021).

Locomotor arrest can also be triggered upon the identification of visual threats (e.g., looming visual stimuli in mice). Such arrest occurs through the activation of competing neuronal paths driven by SuC (Shang et al., 2015, 2018). Activation of the path SuC(PV+ excitatory neurons)-LPTN–LA favors freezing, whereas, activation of the paths SuC(PV+ excitatory neurons)-PBGN-CeA and SuC(PV+ excitatory neurons)-PBGN-dlPAG favors a complex escape-and-freeze behavioral sequence (Figures 2E, 3B). However, the precise neural mechanism underlying the natural balance between PBGN and LTPB is unknown. Furthermore, whether the activation of escape-and-freeze behavior relies on the sequential activation of PBGN-dlPAG and PBGN-CeA-vlPAG for instance also remains elusive.

Moreover, the cerebellum via the FN may oppose the termination of locomotion. As mentioned before, optical activation of afferents from FN at vlPAG (Figure 2A) augments inhibitory control onto Chx10-positive neurons via the activation of D2 dopamine receptors (Vaaga et al., 2020). Vaaga et al. (2020) also showed that optogenetic activation of the Chx10-positive neurons in vlPAG is sufficient to induce freezing. Since activation of vlPAG glutamatergic neurons induces defensive freezing (Tovote et al., 2016), it is probable that the Chx10-positive neurons belong to the same subpopulation of freezing-triggering glutamatergic vlPAG neurons. Hence, increased inhibition of these neurons is expected to reduce freezing while disinhibition does otherwise. Since the FN favors an increase of inhibitory dopaminergic control of vlPAG Chx10 glutamatergic neurons, the cerebellum might also complement the action of the BG direct pathway. However, further studies must be performed to know whether dopamine is released by local TH+ neurons (Vaaga et al., 2020) or by SNc afferents arriving onto vlPAG (Lima et al., 2018).

In summary, the evidence presented in this section indicates that termination of locomotion due to threats (defensive arrest) is supported by the neural circuits residing within hypothalamus (dm/cVMH, PMD), ventral HIPPO, Amygdala (CeA, BLA), and PAG (dl/l/vlPAG) (Figure 3B). The fact that specific pathways between these interconnected subregions have been functionally identified to either directly trigger or more sluggishly promote defensive arrest, indicates that under unperturbed conditions, dynamic contributions of the individual network elements are orchestrated to elicit an adaptive, state-dependent response. Moreover, exploratory arrest is supported by CeA-projecting BLA neurons (Figure 3C). On the other hand, visual-threat driven arrest is mediated by the competing pathways SuC(PV+ excitatory neurons)-LPTN–LA and SuC(PV+ excitatory neurons)-PBGN-dlPAG and PBGN-CeA (Figures 2E, 3B). It remains to be determined how each of these pathways is integrated with downstream motor circuits to generate a coordinated behavioral outcome.

## Discussion

In this review, we described neuronal circuits identified as neural substrates for the state-dependent modulation of locomotion (Figures 1–3). While future studies will likely reveal additional and refine known network elements, in the present review we identified a number of non-exclusive neuronal circuits supporting the different phases of initiation of locomotion, maintenance and arrest/termination.

A brain-wide network involving excitatory circuit elements connecting cortex, midbrain and medullary areas appears to be the common substrate for the initiation of locomotion across different states. In this brain-wide network, the MC and the MLR drive the initial postural adjustment and initiation of locomotion *per se* (Figure 1A). On the other hand, the BG circuits, by implementing action-selection computations, trigger the initiation of goal-directed locomotion (Figure 1C). In addition, the initiation of locomotion is regulated by neuromodulatory circuits residing in the LC, the BF, the hypothalamus and the medulla oblongata (Figures 1B,G). Strikingly, the maintenance of locomotion also requires the interaction of an even larger neuronal network encompassing motor, sensory and associative cortices, as well as the SuC, the cerebellum and Gi (Figure 2). It is conceivable that this is likely due to the need for integration of several information streams, such as sensory and proprioceptive feedback as postural command signals during ongoing locomotion. Nonetheless, the BG seems self-sufficient to drive the bidirectional transition between appetitive and exploratory locomotion, while the regulation of locomotion speed is supported by MLR.

The reviewed evidence indicates that BG and MLR are modulated by both, excitatory as well as inhibitory circuits residing in the hypothalamus, amygdala and PAG to guide the initiation of state-dependent locomotion, i.e., appetitive and defensive locomotion. Complementing these direct influences, GABAergic projections from the LH and the CeA to vl–lPAG are instrumental for the initiation and performance of appetitive locomotion (Figure 1D). Glutamatergic projections from the LH to the vl–lPAG are engaged in the initiation of escape/avoidance behaviors (Figure 2D). Glutamatergic projections from the VMH to the GABAergic neurons in the AHN support the initiation of escape/avoidance behaviors (Figure 1E). Glutamatergic projections from the ventromedial and premammillary hypothalamic nuclei and SuC to d–dlPAG mediate the initiation of aggressive defensive behaviors (Figures 1E,F). Importantly, not only the behavioral context, but also distinct sensory cues establish transient states of emotional valence, which then drive different network elements to elicit adaptive locomotor responses. For example, rapid identification of a visual threat evokes complex dimorphic defensive responses via excitatory neurons in the SuC, which activate the downstream circuits in the parabigeminal nucleus projecting to dlPAG and the CeA (Figure 1F). On the other hand, dimorphic defensive responses are also supported by sensory circuits in the AC projecting to the striatum (D2-SPNs) and to SuC upon the detection of an auditory threat.

Although circuits within the BG seem sufficient to mediate non-defensive yet goal-oriented state transitions, state-dependent initiation, maintenance and termination of locomotion are tightly related to the action of defensive circuits. The transition between non-defensive and defensive behavioral states is strongly reflected by BLA neuronal activity (Gründemann et al., 2019; Fustiñana et al., 2021) and may be functionally complemented by the intra-hypothalamic circuits (Mitrofanis, 2005; Wang et al., 2015), LH-glutamatergic projections to vlPAG, and the SuC-PBGN path to the amygdala (Shang et al., 2018). In addition, the interplay among BG, MLR, and DCN may be the core system for the transition of behavioral states, which require the adaptation of postural muscles. For instance, postural adjustment might be made by activation of ipsilateral synaptic projections from the ZI onto Chx10 Gi glutamatergic neurons, and by the direct communication of glutamatergic MLR neurons to the spinal cord (Bouvier et al., 2015; Ferreira-Pinto et al., 2021).

Locomotor arrest is an important component of defensive emotional states, such as acute anxiety, which are mediated via a network of survival circuits involving hypothalamus, amygdala and PAG, connecting to medullary premotor centers (Figure 3A). Activation of hindbrain GiA/LPGi/GiV/Gi glycinergic neurons and the bilateral activation of Chx10 Gi glutamatergic neurons can all also directly trigger gait disruption resulting in locomotor arrest. On the other hand, behavioral arrest driven by decision-making processes relies more on a complex interaction between the BLA (Botta et al., 2020), the BG-MLR circuits (Kravitz et al., 2012; Picciotto et al., 2012; Roseberry et al., 2016; Moehle et al., 2017; Ferreira-Pinto et al., 2018) and PAG. While these findings suggest that PAG circuits constitute major regulatory units for state-dependent locomotion, the precise mechanisms on how this is translated into specific motor programs are poorly understood. Conceptually, the PAG plays multiple roles in the control of state-dependent locomotion by integrating coherent information from the SuC, the amygdala, the hypothalamus, and the cortex, as well as, calculating threat probability (Wright and McDannald, 2019) and delivering an adaptive executive command to downstream premotor circuits. In line, neuroanatomical data support a possible routing of integrated state-dependent information from the PAG to MLR to drive locomotion (Caggiano et al., 2018).

### Integration of Supraspinal and Spinal Cord Circuits

While during the last decade, research has greatly promoted our understanding of supraspinal circuits involved in locomotion, how specific supraspinal circuits communicate with the spinal cord, where these pathways converge, and which circuit elements are shared remains poorly understood. Nonetheless, recent evidence points toward several integration centers throughout the neural axis. For example, one described locomotion initiation pathway resides in the MLR–LPGi glutamatergic circuit, while MLR-spinal cord glutamatergic pathway controls postural adjustments which are required for proper locomotion initiation. Moreover, maintenance of rhythmic locomotion and locomotion termination rely on glutamatergic and glycinergic circuits located in Gi, LPGi, and GiA, which receive differential synaptic input from DLR, CLR, and MLR. Since activation of GiV glycinergic neurons produces atonia, these neurons help to keep a relaxed state of skeletal muscles during sleep (Garcia et al., 2018). Furthermore, the control of gait function requires the orchestrated interaction of neural circuits residing in sensory brain areas, associative-limbic areas, and attentional/reward areas with defensive circuits providing direct control of motor responses and autonomic functions. In the neural concert of gait function, it is conceivable that the hypothalamus, the amygdala and PAG play a central role by funneling emotional information into the MLR. In this concert, the BG together with the cerebellum will drive cognitive goal-oriented locomotion with adaptive postural adjustment which may support the transition among several locomotion states.

### Translation of Circuit Mechanisms From Animal to Human Brains

Unsurprisingly, there is a large overlap of circuits for state-dependent control of locomotion. However, while it is clear that gait coordination on the mechanical level needs temporally precise circuit interactions, such as those proposed for central pattern generators, the multi-level interaction of higher-order centers for state-dependent modulation of locomotion is striking. In the modern era of circuit neuroscience, with its cell-type and projection-specific tools as well as complex behavioral and kinematic analyses, we have just begun to understand the complexity of the control of locomotion. As a consequence, much of the detailed findings obtained in animal models remain to be translated into research approaches in humans. Emotional states such as appetite, anger and fear are important drivers of volitional movements in humans, and there is abundant evidence that these basic emotional states involve similar brain regions across mammalian species. But do the neuronal pathways and circuits, based on findings from animal studies, account for the modulation of initiation, maintenance, and termination of locomotion, as well as postural control also in humans? Unfortunately, lower resolution so far limits the investigation of cell-type specific circuits in the living human brain, a barrier that will be hard to overcome in the near future. Nonetheless, findings from animal models on the level of brain (sub-)regions support the design of testable hypotheses to be pursued via functional neuroimaging approaches in humans. To push the translational relevance of research in animal models, efforts to integrate small-scale circuit findings on the level of cell types with the larger networks across brain regions (Mace et al., 2013; Cardin et al., 2020; Markicevic et al., 2021) should be undertaken. Furthermore, the development of similar behavioral paradigms and use of common readouts as well as analyses present promising avenues for successful cross-species translation. Clearly, basic insights into circuit function can inform and help refine established interventional strategies such as deep-brain electrical stimulation (DBS) or new approaches using ultrasound or electromagnetic energy to manipulate local brain activity.

### The Handling of Pathological Gait Dysfunctions

Clinical evidence shows that gait dysfunction occurs upon the damage of different brain areas such as the cerebellum, the BG circuits including putamen, internal globus pallidus and external globus pallidus, the primary MC, the brainstem, the midbrain/tegmentum, the corpus callosum and the parasagittal white matter (Nutt et al., 2011; Fasano et al., 2017). Moreover, imaging alterations in networks for executive attention, including frontal lobe, and networks for emotional processing, including amygdala, have been linked to locomotor dysfunction, such as FoG in PD (Fasano et al., 2015; Gilat et al., 2018). Since FoG in PD patients is understood as the inability to produce effective forward stepping, any emotional or cognitive restraint preventing or delaying the activation of neural circuits underlying the initiation of locomotion may favor the emergence of FoG. Although clear links between anxiety and motor symptoms in PD such as FoG have been established (Martens et al., 2016), their interplay is mechanistically not understood. Our review introduces a more holistic perspective, thereby identifying points of interaction within the larger neuronal network, where (pre-) motor circuits mediating termination of locomotion and the “limbic” circuits mediating emotional states such as fear and anxiety converge. Further research using selective optogenetics, transsynaptic tracing, calcium imaging and electrophysiology *in vivo* and *ex vivo* needs to be done in animal models of PD and ataxia to better understand the functional alterations of the neural network dynamics and synapses in different locomotor regions.

The clinical standard treatment of PD symptoms, dopamine replacement, has been proven relatively ineffective to ameliorate gait dysfunction. Non-pharmacological alternative procedures to mitigate gait dysfunction in advanced PD patients have been implemented. Many of these procedures rely on DBS targeting STN, GPi and PPN (PPT) or less invasive spinal cord electrical stimulation (Peppe et al., 2010; Welter et al., 2015; Samotus et al., 2018; Hartmann et al., 2019; He et al., 2020). However, the mechanisms of action remain largely unclear as the outcomes are highly variable depending on the assessed gait parameter, body parts (e.g., legs, arms or trunk postural muscles), stimulation frequency, targeted brain area or whether the stimulation is combined or not with pharmacological treatment. A major caveat of DBS, besides its invasiveness, is that electrical stimulation does not discriminate among neuronal cell types, which may generate major alterations in the natural performance of unspecific neighbor networks. For example, direct stimulation of PPN may dampen volitional locomotion either via activation of a counterbalancing feed-back cholinergic control on the BG direct pathway or via increased PPN glutamatergic drive to BG output regions. Moreover, DBS in PPN may alter glutamatergic descending pathways conveying postural and locomotor commands to the spinal cord and may activate DCN with distinct cognitive and motor functional roles. However, combinations of pharmacological receptor blockade and DBS could in principle increase the pathway selectivity of electrical stimulation (Creed et al., 2015). As it becomes increasingly clear that the network functions underlying control of locomotion and gait are highly state-dependent, more dynamically adjusted DBS, such as closed-loop approaches involving concomitant recordings and stimulation, could present more precise and potentially more effective network “retuning” action.

Overall, part of the tremendous complexity of the state-dependent circuitry regulating locomotor functions can be explained by the demand to react to various environmental changes and challenges. This requires dynamic integration of fast behavioral responses to specific cues with evaluation of varying contexts, constituting a selection pressure that drove step-wise evolution of interactive neuronal circuit modules serving ever-increasing flexibility of adaptive behavioral repertoires. However, the highly interconnected, and inter-dependent function of these networks thereby became vulnerable for dysregulation within individual modules. Consequently, from a modern systems neuroscience perspective, motor dysfunctions reflect network diseases, so called circuitopathies, which take into account the regulation of locomotor functions by higher-order states.

## Author Contributions

AP-A, NW, ME, and PT wrote the review article. All authors contributed to the article and approved the submitted version.

## Conflict of Interest

The authors declare that the research was conducted in the absence of any commercial or financial relationships that could be construed as a potential conflict of interest.

## Publisher’s Note

All claims expressed in this article are solely those of the authors and do not necessarily represent those of their affiliated organizations, or those of the publisher, the editors and the reviewers. Any product that may be evaluated in this article, or claim that may be made by its manufacturer, is not guaranteed or endorsed by the publisher.

## Abbreviations

AC: auditory cortex
AHN: anterior hypothalamic nucleus
BF: basal forebrain
BG: basal ganglia
BLA: basolateral amygdala
CCK: cholecystokinin
CeA: central amygdala
Chx10: CEH10 homeodomain-containing homolog
CLR: cerebellar locomotor region
CNF: cuneiform nucleus
CTX: cortex
DBS: deep brain stimulation
DCN: deep cerebellar nuclei
DLR: diencephalic locomotor region
DN: dentate nucleus
FN: fastigial nucleus
FoG: freezing of gait
GABA: gamma-aminobutyric acid
GAD2: glutamate decarboxylase 2
Gi (A, V): gigantocellular nucleus (alpha part, ventral part)
GP (e, i): globus pallidus (external, internal)
IN: interpositus nuclei
KARs: kainate glutamate receptors
LA: lateral amygdala
LC: locus coeruleus
LH: lateral hypothalamus
LPGi: lateral paragigantocellular nucleus
LPTN: lateral-posterior thalamic nucleus
MC: motor cortex
MLR: mesencephalic locomotor region
MN: mammillary nucleus
mRt: mesencephalic reticular region
NAcSh: nucleus accumbens shell
PAG (d, dl, l, vl, v): periaqueductal gray matter (dorsal, dorsolateral, lateral, ventrolateral, ventral)
PBGN: parabigeminal nucleus
PCRt: parvicellular reticular formation
PD: Parkinson’s disease
PFC (m, dm): prefrontal cortex (medial, dorsomedial)
PMD: premammillary nucleus of the hypothalamus
PPN: pedunculopontine tegmental nucleus
PV: parvalbumin
REM: rapid eye movement
SN (c, r): substantia nigra (compacta, reticulata)
SPN: striatal projection neuron (MSN)
STN: subthalamic nucleus
SuC: superior colliculus
TH: tyrosine hydroxylase
vHIPPO: ventral hippocampus
VMH (c, vl, dm): ventromedial hypothalamus (central, ventrolateral, dorsomedial)
ZI: zona incerta.
